# Microbiota and gut ultrastructure of *Anisakis pegreffii* isolated from stranded cetaceans in the Adriatic Sea

**DOI:** 10.1186/s13071-019-3636-z

**Published:** 2019-07-30

**Authors:** Ivona Mladineo, Jerko Hrabar, Anamarija Vrbatović, Sanja Duvnjak, Tomislav Gomerčić, Martina Đuras

**Affiliations:** 1 Laboratory of Aquaculture, Institute of Oceanography and Fisheries, Split, Croatia; 2 Laboratory of Zoonotic Bacteria and Molecular Diagnosis of Bacterial Diseases, Department of Microbiology and Parasitology, Croatian Veterinary Institute, Zagreb, Croatia; 3 Faculty of Veterinary Medicine, Zagreb, Croatia

**Keywords:** *16S* rRNA gene sequencing, *Anisakis pegreffii*, Microbiota, Striped dolphin (*Stenella coeruleoalba*), TEM

## Abstract

**Background:**

Inferring the microbiota diversity of helminths enables depiction of evolutionarily established ecological and pathological traits that characterize a particular parasite-host interaction. In turn, these traits could provide valuable information for the development of parasitosis control and mitigation strategy. The parasite *Anisakis pegreffii* (Nematoda: Anisakidae) realizes the final stage of its life-cycle within gastric chambers of aquatic mammals, causing mild-to-moderate granulomatous gastritis with eosinophilic infiltrate, to severe ulcerative gastritis with mixed inflammatory infiltrate, often associated with bacterial colonies. However, its interaction with the host microbiota remains unknown, and might reveal important aspects of parasite colonization and propagation within the final host.

**Methods:**

MySeq Illumina sequencing was performed for the *16S* rRNA gene from microbiota isolated from larvae, and uterus and gut of adult *A. pegreffii* parasitizing stranded striped dolphins (*Stenella coeruleoalba*). To assess the potential presence of *Brucella ceti* within isolated microbiota, *Brucella*-targeted real-time PCR was undertaken. In addition, TEM of the gastrointestinal tract of the infective third-stage (L3) and transitioning fourth-stage larvae (L4) was performed to characterize the morphological differences and the level of larval feeding activity.

**Results:**

In total, 230 distinct operational taxonomic units (OTUs) were identified across all samples (*n* = 20). The number of shared taxa was lower than the number of taxa found specifically in each parasite stage or organ. The dominant taxon was *Mycoplasmataceae* (genus *Mycoplasma*) in the gut and uterus of adult *A. pegreffii*, whereas *Fusobacteriaceae* (genus *Cetobacterium*) was the most abundant in 40% of larvae, alongside *Mycoplasmataceae*. No *B. ceti* DNA was detected in any of the microbiota isolates. TEM revealed differences in gut ultrastructure between L3 and L4, reflecting a feeble, most likely passive, level of feeding activity in L3.

**Conclusions:**

Microbiota from L3 was more related to that of the gut rather than the uterus of adult *A. pegreffii*. Taxa of the larval microbiota showed qualitative and quantitative perturbations, likely reflecting the propagation through different environments during its life-cycle. This suggests an ontogenetic shift in the alpha and beta diversity of microbial communities from uterus-derived towards cetacean-derived microbiota. Although TEM did not reveal active L3 feeding, microbiota of the latter showed similarity to that of an actively feeding adult nematode.

**Electronic supplementary material:**

The online version of this article (10.1186/s13071-019-3636-z) contains supplementary material, which is available to authorized users.

## Background

Ascaridoid nematodes from genus *Anisakis* complete their life-cycle in the marine environment, with crustaceans, mostly euphausiids, as the first intermediate hosts, fish and cephalopods as paratenic hosts, and cetaceans and pinnipeds as the final hosts, where they reach the adult stage and reproduce [1]. During the development from the first (L1) to early third larval stage (L3), the larvae are sheltered from unpredictable environmental conditions [2] in the intermediate hosts that can spatiotemporally extend their life and support their growth by providing a valuable nutrient-rich environment. After moulting to the infective L3 stage, some nematodes such as *Anisakis* spp. persist in the host without essential development and growth (paratenesis) [3]. This feature evolved as a consequence of the feeding preference of the final host toward a new prey, which feeds upon the intermediate host, and consequently becomes a paratenic host. Anderson [4] suggested that the phenomenon was likely crucial for the survival of many nematode parasites during the evolution of order Carnivora from ancestors. More importantly, the existence of paratenic hosts extends the number of encounters between host and parasite, enabling the accumulation and extended lifespan of the latter. It is widely accepted that *Anisakis* spp. L3 represents a non-feeding stage; however, it remains unknown whether they utilize their embryonically stored nutrients, enter a state of hypobiosis, or absorb minimal amounts through cuticle when inside the paratenic host, as described for other larval nematodes [5] such as the filarial nematode *Brugia pahangi*, which exchanged its non-functional gut for trans-cuticular absorption of nutrients [6]. *In vitro* experiments have reported that after completion of *Anisakis* spp. moulting into stage four larvae (L4), the larval intestine becomes functional [7], subsequently enabling its massive colonization by the microbiota inhabiting the gastric chambers of the final host.

Associations between microbes and the parasitic nematodes they inhabit have garnered interest because such interactions shape the nematode biology, ecology and its survival in general (reviewed in [8, 9]). However, these associations in turn also affect the host in which the nematode propagates, through feeding and reproduction (reviewed in [10]).

*Anisakis* spp. are zoonotic helminths, and humans can become accidental hosts by consumption of raw or inadequately thermally treated fishery products contaminated with live *Anisakis* spp. larvae. Infective L3 represent a public health risk for a zoonotic disease known as anisakidosis or anisakiasis [11], with an added theoretical risk of propagation of cetacean bacterial pathogens to human beings. Although a typical acute onset of anisakiasis in humans is usually caused by a single migrating larva, sometimes inferred only as an accidental finding during laparoscopy [12], recently, two cases in Europe have reported massive *Anisakis* spp. infection. No histopathological changes had developed in either of the two cases, as the larvae were immediately extracted through gastroendoscopy, representing a tally of more than 200 [13] and 140 larvae [14], respectively.

To investigate novel aspects of *Anisakis* spp. biology that may help improve our understanding of the parasite-host interactions, we (i) explored the bacterial biodiversity in adults and third-stage larvae (L3) of *Anisakis pegreffii* isolated from Adriatic stranded striped dolphins (*Stenella coeruleoalba*) using *16S* rRNA gene sequencing; (ii) assessed the potential presence of *Brucella ceti* within the microbiota isolated from adult *A. pegreffii*; and (iii) morphologically characterized the ultrastructure of the gastrointestinal tract of *A. pegreffii* infective third-stage larvae (L3), relative to the tract of functionally feeding fourth-stage larvae (L4), using transmission electron microscopy (TEM).

## Methods

### Stranded cetaceans and sampling of *Anisakis* sp

*Anisakis* sp. were sampled from two animals: a young adult female striped dolphin (*Stenella coeruleoalba*), code #411, found stranded on 15 June 2017 in Opatija (total length: 196 cm; total weight: 57 kg; decomposition code: fresh), and an adult aged male striped dolphin, code #419, found stranded on 5 September 2017 in Stari Grad, Hvar (total length: 213 cm; total weight: 67 kg; decomposition code: fresh).

The animals were necropsied following standard protocol [15]. Adult *Anisakis* sp. were isolated from dolphin #411, whereas L3 and L4 *Anisakis* sp. larvae (the latter used only for TEM analysis; adults were not present) were isolated from dolphin #419. Both samples were taken from the fore stomach (first gastric chamber) and immediately frozen at − 80 °C for further microbiota DNA isolation.

### *Anisakis*-associated microbiota DNA extraction

Sample preparation for DNA extraction followed a previous study [16]. Prior to DNA isolation, all the required equipment (stereomicroscope, dissecting tools, thermo-shaker, pipettes) were cleaned in Ecocid®S (Krka d.d., Novo Mesto, Croatia) and UV-irradiated, and moved into the laminar flow cabinet, where all steps of the DNA isolation protocol were carried out, except centrifugation. The samples were thawed and washed with 100 mM levamisole in M9 buffer, incubated for 1 h in M9 containing 100 mM levamisole and 100 mg/ml gentamicin, and then washed with a levamisole solution to remove the gentamicin. Adult parasites were then dissected by longitudinal section, and the gastrointestinal system (from oral to anal opening) and gravid uterus were individually sampled from five adult females. As *Anisakis* sp. L3 has no functional gut or gonads, the whole body (n = 10) was homogenized, after incubation, in a levamisole-gentamicin solution. The adult gut and uterus samples were manually homogenized by pestle, whereas whole L3 were homogenized using sterile beads in a MagnaLyser (Roche, Basel, Switzerland). A PureLink Microbiome DNA purification kit for purification of high-quality microbial and host DNA from stool samples (Invitrogen, Carlsbad, CA, USA) was used for DNA extraction. Quality and quantity of the extracted DNA were assessed using a GenovaNano spectrophotometer (Jenway, Staffordshire, UK).

### Microbiota DNA library preparation and sequencing

The extracted DNA samples (*n* = 20), divided into three groups (10 larvae, five adult gut and five adult uterus samples) were sent for commercial DNA library preparation and sequencing to MR DNA (Shallowater, TX, USA; http://www.mrdnalab.com,). In brief, the *16S* rRNA gene V4 variable region was amplified by PCR primers 515/806 with barcode on the forward primer using the HotStarTaq Plus Master Mix Kit (Qiagen, Hilden, Germany) and the following conditions: 94 °C for 3 min, followed by 28 cycles of 94 °C for 30 s, 53 °C for 40 s, and 72 °C for 1 min, and a final elongation step at 72 °C for 5 min.

The PCR products were verified using 2% agarose gel, following which the samples were pooled together in equal proportions based on their molecular weight and DNA concentrations, purified using calibrated Ampure XP beads, and used for DNA library preparation following the Illumina TruSeq DNA library preparation protocol. Sequencing (250 bp paired-end reads) was performed on a MiSeq following the manufacturer’s guidelines, and the sequence data were processed using Exaltum analysis pipeline (Exaltum, Zagreb, Croatia; http://exaltum.eu). Details of the dataset are shown in Additional file 1: Table S1.

### Microbiota data analysis

Quality control of raw unmultiplexed fastq files and a total of 1,918,235 sequences was carried out with FastQC, to assess the sequencing quality. No sequences were filtered out due to poor quality.

The analysis pipeline was based on the QIIME2 [17] workflow (version 2018.4.0), and included the following steps. First, removal of adapters and demultiplexing based on barcode sequence was carried out with cutadapt [18]. Secondly, filtering and denoising were done to remove the internal sequencing errors and chimeric sequences (DADA2; [19]). The final read counts after all pre-processing steps are outlined in Additional file 1: Table S2. Thirdly, identification of distinct sequences across samples was carried out (DADA2; [19]). A total of 230 distinct sequence features were identified across 20 samples (Additional file 2: Figure S1), with a total frequency of 502,271. The maximum and minimum frequency per feature was 84,702 and 2.0, respectively, while the maximum and minimum frequency per sample was 34,469 and 11,497, respectively. Fourthly, multiple alignment of sequence features was carried out with mafft [20], wherein non-informative sites were masked, and a rooted tree was constructed with FastTree [21]. Fifthly, alpha diversity, describing the within-sample phylogenetic richness using Shannon diversity, Pielou’s evenness and Faith’s phylogenetic diversity index, was computed [22]. The sample sequences were subsampled (rarefied) to two depths: minimal (11,497; 45.8% sequences), which included all samples; and optimal (20,000; 71.7% sequences), which eliminated two larval samples, 419h and 419j. The effect of selected sequence depth on measured indices was evaluated for each index by plotting the index calculated per sample at two rarefaction levels (Additional file 3: Figure S2). The alpha-rarefaction indices were analyzed using Kruskal–Wallis test to determine significant differences between stage and pairwise differences among the three sample groups. Sixthly, beta-diversity, measuring differences between samples and the community consistency across the entire experiment, was evaluated using Jaccardʼs, Bray–Curtis, unweighted and weighted UniFrac distances.

Statistical comparison of bacterial communities among the three groups was performed using PERMANOVA (999 permutations, *P* = 0.001), principal components analysis (PCA), principal coordinates analysis (PCoA) and canonical correspondence analysis (CCA) [23], which was followed by operational taxonomic unit (OTU) assignment. Finally, differential abundance was calculated using the ALDEx method proposed by Fernandes et al. [24].

The sequence features were taxonomically assigned using the Silva database [25], with 99% clustering cutoff and QIIME2-trained classifier for *16S* V4 primers, and compiled into each taxonomic level into “counts” (actual number of sequences) and “percentage” (proportion of sequences within each sample that map to the designated taxonomic classification) files.

### Data deposition

The obtained sequences were deposited in BioProject database (https://www.ncbi.nlm.nih.gov/sra), ID PRJNA512895, with consecutive accession numbers from SAMN10690278-SAMN10690297.

### *Brucella-*targeted RT-PCR

Although dolphin samples taken for bacteriological isolation of *Brucella* spp. (data not shown) tested negative, a subsample of *Anisakis* adults and larvae microbiota DNA was isolated as previously described and used as the template for *Brucella*-targeted real time PCR (RT-PCR) [26].

### *Anisakis* sp. molecular identification

An aliquot of extracted DNA was used to identify *Anisakis* genotypes using the mitochondrial cytochrome oxidase 2 (*cox*2) (~ 600 bp) and internal transcribed spacer (ITS) locus (~ 1000 bp, spanning through ITS1, *5.8S* rRNA gene and ITS2). The latter amplicons were afterwards digested by restriction endonuclease *Hinf*I (Promega, Madison, WI, USA) for restriction fragment length polymorphism analysis (RFLP-PCR) [27]. This step was necessary to exclude species other than *A. pegreffii*, potentially introduced into the striped dolphins while migrating from the Mediterranean.

### Ultrastructure of the gut of L3 and L4 larvae

For transmission electron microscopy, the L3 larvae were cut with 1 mm biopsy punches (Integra Miltex, Plainsboro, NJ, USA) into five parts, corresponding to the anterior end with pharynx, ventriculus, anterior part of intestine, posterior part of intestine, and the tail. Each section was placed on a metal disk and high-pressure frozen in the presence of 20% BSA in EM PACT2 (Leica Microsystems, Vienna, Austria). Afterwards, the sections were subjected to freeze substitution in 2% OsO_4_ in acetone at − 90 °C for 96 h. The temperature was then raised to − 20 °C (5 °C/h) and kept at − 20 °C for 24 h. Finally, the temperature was raised to 4 °C (3 °C/h) and maintained at 4 °C for additional 20 h.

The samples were then washed in acetone (three times, 15 min each) and infiltrated with 25, 50 and 75% mixtures of Low Viscosity Spurr resin (SPI Chem, West Chester, PA, USA) and anhydrous acetone, for 1 h each. The samples were left in pure resin overnight, transferred to embedding moulds and polymerized for 48 h at 60 °C. L4 larvae were cut into same sections as L3 larvae using two sharp blades, and fixed overnight in 4% paraformaldehyde in 0.1 M phosphate buffered saline (PBS) at 4 °C. The samples were then washed in PBS (three times, 15 min each), postfixed in 2% aqueous OsO_4_ for 2 h at room temperature, and dehydrated in a graded series of acetone solutions (30–100%), with 15 min at each step. Resin infiltration and embedding was performed as described above.

Semi-thin sections were cut at 0.5 μm thickness, stained with 1% toluidine blue, and observed under a light microscope for orientation. Ultrathin sections were cut at 0.07 μm thickness, placed on formvar coated single slot grids, contrasted in ethanolic uranyl acetate (30 min) and lead citrate (20 min), and observed under a JEOL 1010 TEM (JEOL, Akishima, Tokyo, Japan) operating at an accelerating voltage of 80 kV. Images were captured with Mega View III camera (Olympus Soft Imaging Solutions GmbH, Münster, Germany), and assembled and annotated in PhotoShop CS5 software (Adobe Systems, San Jose, CA, USA).

## Results

### *Anisakis pegreffii* microbiota

Sequencing of *16S* rDNA of microbiota from the uteri and gastrointestinal tracts of five adult and ten third-stage *A. pegreffii* larvae parasitizing Adriatic-stranded striped dolphins (*S. coeruleoalba*) resulted in total of 1,918,235 raw sequences (forward and reverse reads, sequence length 35–251 bp, mean GC content 48%). After removal of chimera reads and other non-bacterial sequences, a total of 1,247,073 sequences (mean ± SE = 62,353.65 ± 51,161.74; minimum = 39,376, maximum = 259,839) were retained.

After rarefaction at 20,000 reads per sample (optimal depth), two larval samples (419h and 419j) were eliminated due to low sequence count, while retaining 71.7% of the total reads. The minimal depth set at 11,497 included all the samples, retaining 45.8% of the total reads across all samples. No significant effect of sequencing depth on the results of the Shannonʼs and Faith’s diversity indices or Pielou’s evenness was observed, indicating that the samples were sufficiently covered and the results were robust.

Similarly, a representative graph of alpha rarefaction plots (observed OTUs *vs* sampling depth) demonstrated that the sampling for this experiment was sufficient (Additional file 4: Figure S3).

In total, 230 distinct OTUs (total frequency 502,271) were identified across all samples, suggesting that the three sample groups (larvae, adult uterus, adult gut) were specific in terms of their microbial content. The number of shared taxa was lower than the number of taxa found specifically per stage/organ (Additional file 2: Figure S1).

A small number of *A. pegreffii* samples encompassed a large number of OTUs, e.g. nine and fewer *A. pegreffii* samples had 209, or 90.9%, of the total 230 bacterial OTUs, whereas a single sample contained 166, or 72.2%, of the total OTUs. In contrast, only two, or 0.87%, of the total OTUs were observed in all 20 *A. pegreffii* samples.

Rarefaction analysis showed that the richness and diversity varied among microbiota within the three *A. pegreffii* groups, while rarefaction curves for all samples reached the plateau, indicating that rare bacterial taxa were successfully recovered (Additional file 4: Figure S3). The trend of each alpha diversity index per developmental stage (larva, adult) and sample (larva, gut, uterus) is shown in Additional file 5: Figure S4. The average microbiota diversity was 3.846 in larvae, 3.859 in adult gut and 3.339 in adult uterus, while the microbiota evenness was 2.952 in larvae, 4.537 in adult gut and 5.141 in adult uterus. The statistical difference in Pielou’s evenness and Faith’s diversity (rarefaction 20 k reads) between microbiota of *Anisakis* developmental stage/organ, evaluated by Kruskal–Wallis test (*H* = 12.41, *df* = 17, *P* < 0.001), is shown in Table 1, showing that the only difference between the microbiota of adults and larvae was in terms of community evenness.

**Table 1:** Difference in Pielou’s evenness (lower diagonal) and Faith’s diversity (upper diagonal, in bold) among microbiota (rarefaction at 20 k reads) isolated from adult uterus and gut, and L3 larvae, of *A. pegreffii* parasitizing striped dolphins (*Stenella coeruleoalba*) stranded in the Adriatic Sea, tested using the Kruskall–Wallis test (*P < 0.001)

	Adult	Larva		Uterus	Gut	Larva
Adult	0	**0.00182**	Uterus	0	**0.916815**	**0.003415**
Larva	0.000734*	0	Gut	0.117185	0	**0.028108**
			Larva	0.003415	0.008415	0

Statistical differences (pairwise PERMANOVA, *Pseudo*-*F* = 5.01, *P* < 0.001) in beta diversity indices (Jaccardʼs distance, Bray–Curtis, unweighted Unifrac distance, weighted Unifrac distances) between the microbiota of *A. pegreffii* developmental stages and adult *A. pegreffii* organs are shown in Table 2, where the only difference between the microbiota of adult gut and larvae, though marginal, was in terms of Jaccardʼs distance dissimilarity index. Two-dimensional relationships between beta diversity indices of microbiota of adult gut and larvae, analyzed through PCA, PCoA and CCA, are shown in Additional file 6: Figure S5.

**Table 2:** Part **a** shows difference in Jaccardʼs distance (lower diagonal) and Bray-Curtis distance (upper diagonal, in bold) and part **b** shows difference in unweighted Unifrac distance (lower diagonal) and weighted Unifrac distance (upper diagonal, in bold). Differences are calculated among the microbiota (rarefaction at 20 k reads) isolated from adult uterus and gut, and L3 larvae, of *A. pegreffii* parasitizing striped dolphins (*Stenella coeruleoalba*) stranded in the Adriatic Sea, tested by pairwise PERMANOVA (*P < 0.001)

a	Uterus	Gut	Larva	b	Uterus	Gut	Larva
Uterus	0	**0.128**	**0.008**	Uterus	0	**0.026**	**0.002**
Gut	0.051	0	**0.017**	Gut	0.041	0	**0.035**
Larva	0.003	0.001*	0	Larva	0.015	0.013	0

**Fig. 1:**
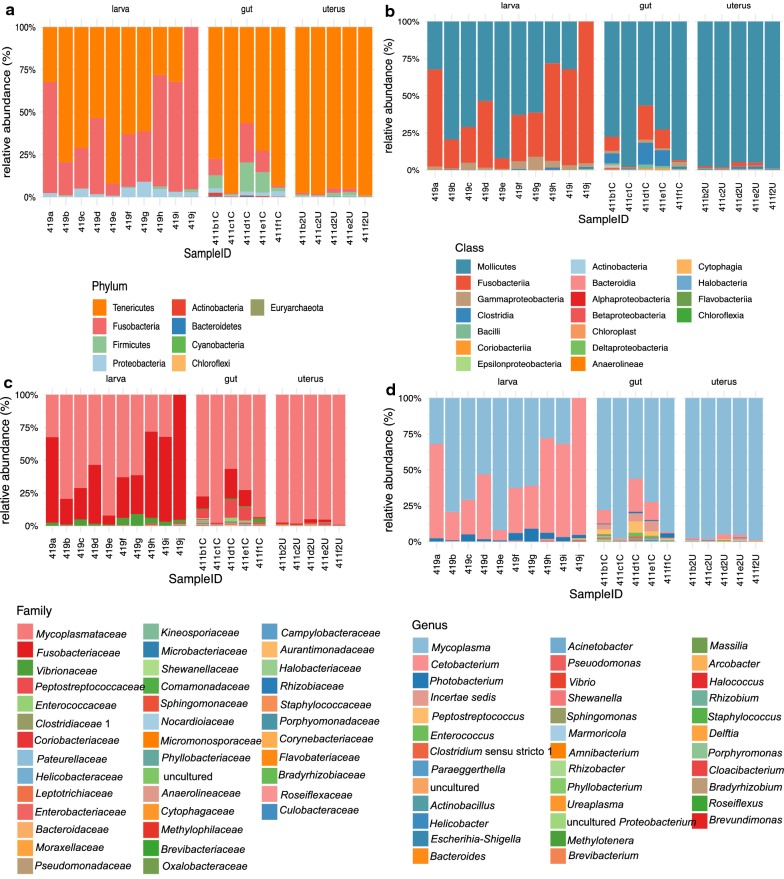
The relative abundance of microbiota taxa at **a** phylum; **b** class; **c** family; and **d** genus level, identified in the *Anisakis pegreffii* infective third-stage larvae (larva), adult uterus (uterus) and adult gut (gut), obtained from striped dolphins (*Stenella coeruleoalba*) stranded in the Adriatic Sea

A total of nine bacterial phyla, encompassing 18 classes, were identified in *A. pegreffii* specimens of which the majority of unassigned ones (12.94%) were observed in adult uterus samples. The predominant phylum was Tenericutes in adult uterus (97.34%) and gut (97.39%) samples, whereas the dominant phylum in larval samples was Fusobacteria (92.23%). The relative abundance of these taxa varied among groups (Fig. 1). The dominant taxon was *Mycoplasmataceae* in adult gut and uterus, whereas in 40% of larvae, *Fusobacteriaceae* was the most abundant, alongside *Mycoplasmataceae*. This was reflected at the genus level, as *Mycoplasma* dominated in all three *Anisakis* groups, except in 40% of the larvae, where *Cetobacterium* was the most abundant genus. Differential taxonomy analysis of the microbiota of *A. pegreffii* adult gut and larvae is shown at the level of phylum and class in Additional file 7: Figure S6, at the level of family in Additional file 8: Figure S7, and at level of genus in Additional file 9: Figure S8.

### *Anisakis pegreffii* molecular identification

Inferred by mtDNA *cox*2 locus, all 15 individuals belonged to *A. pegreffii*, while RFLP-PCR detected a single *A. pegreffii* × *A. simplex* (*sensu stricto*) recombinant genotype from the larval sample (7%).

### *Brucella-*targeted RT-PCR

RT-PCR of the tested samples was negative for the presence of *Brucella* sp. DNA, as no *Brucella* specific IS711 element was identified.

### Ultrastructure of the gastrointestinal tract of L3 and L4

TEM analysis of L3 and L4 alimentary tract showed that the two stages have similar general ultrastructure, although with several notable differences.

**Fig. 2:**
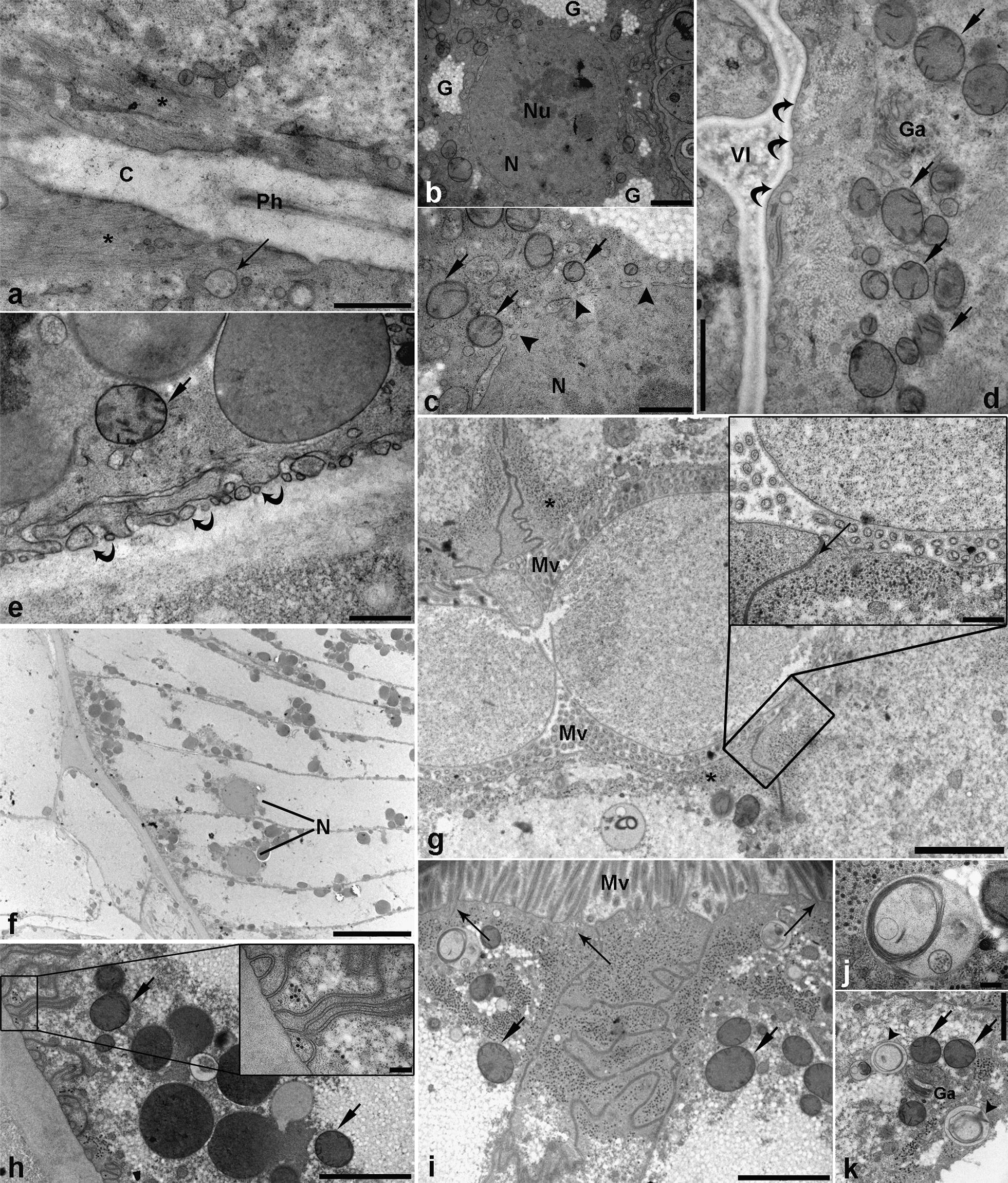
Representative electron micrographs of third-stage *Anisakis pegreffii* larva gut. **a** Pharynx with uniformly dense cuticle (C) lining the pharyngeal lumen (Ph). Thin electron-lucent muscle fibres (asterisk) are radiating from the cuticle. Arrow indicates the multivesicular body (MVB). **b** Nucleus of pharyngeal cell (N) with prominent nucleolus (Nu), surrounded by patches of granulated material consistent with glycogen. **c** Detailed view of the pharyngeal cell nucleus with nuclear pores (arrowheads) and crista-type mitochondria in perinuclear space (arrows). **d** Ventriculus of L3 with fine electron-lucent content in ventricular lumen (Vl). Apical membrane of ventricular cells lacks brush border (curved arrows), while subapically, crista-type mitochondria (arrows) and single Golgi apparatus (Ga) are seen. **e** Basal part of the ventricular cell, with electron-lucent vesicles and single mitochondria (arrow). Note the small invaginations of the basal membrane with vesicles close to it (curved arrows). **f** Spindle-shaped enterocytes with basally located nuclei (N). **g** Intestinal lumen of L3 with two large vesicles circumscribed by double membrane and filled with finely granulated electron-lucent content. Microvilli (Mv) are seen in cross section. Note the subapical cytoplasm filled with numerous electron-dense tubular structures (asterisks). Insert: detailed view of a vesicle inside the intestinal lumen and apical part of two cells separated by tight junction (arrow). Subapically, abundant electron-dense tubular structures are seen. **h** Basal part of an enterocyte with crista-type mitochondria (arrows), and large, electron-dense vesicles consistent with yolk. Note the elaborate invaginations of the basal membrane. Insert: detailed view of basal membrane invaginations, with electron-dense tubular structures. **i** Subapical complex junction separating two cells, with mitochondria (arrows) and vesicles adjacent to lateral membranes. Note the small rootles forming a terminal web (long arrows) extending from the basal ends of microvilli (Mv). **j** Large MVB with a granule, surrounded by multi-layered membranes resembling myelin sheath. **k** Subapical cytoplasm with mitochondria (arrows), Golgi apparatus (Ga), and granules surrounded by multi-layered membranes (arrowheads). *Scale-bars*: **a**, **c**, **e**, insert in **g**, 500 nm; **b**, **d**, **k**, 1 µm; **f**, 10 µm; **g**, **h**, **j**, 2 µm; **i**, insert in **h**, 200 nm

The pharynx of L3 has a triradiate lumen, lined with uniform, electron-dense cuticle (Fig. 2a), contrasting with the cuticle secreted on the nematode surface, which is composed of multiple layers of different electron density. Pharynx surrounding the cells had electron-lucent cytoplasm with scant vesicles and multivesicular bodies (MVBs) concentrated adjacent to the apical (luminal) cell membrane. Bundles of muscle fibres were also seen, radiating from the cuticle lining the pharynx (Fig. 2a). Basally located large nuclei, with prominent nucleoli and nuclear pores, were surrounded by patches of coarsely granulated cytoplasm resembling glycogen deposits (Fig. 2b). Crista-type mitochondria of different sizes and with scant cristae were observed, predominantly in the perinuclear region (Fig. 2c). Free ribosomes were diffusely dispersed apically in cytoplasm or interspersed between perinuclear mitochondria. The pharynx extended further, to a short ventriculus with triradiate lumen when collapsed, which appeared to be partly filled with diffuse electron-lucent content (Fig. 2d).

Cells forming the ventriculus had a smooth apical membrane, lacking a brush border. Mitochondria with scant cristae were seen subapically. Moreover, numerous amorphous tubular electron-dense structures were present interspersed between the mitochondria. Ribosomes were seen as either free organelles or attached to rough endoplasmic reticulum (RER). Basally, the cytoplasm of ventricular cells contained numerous vesicles of various sizes, and the membrane was invaginated, neighbouring numerous small vesicles (Fig. 2e).

The ventriculus continued into intestine with triradiate lumen when collapsed, and was lined with cellular epithelium. A monolayer of spindle-shaped enterocytes encompassed the same granulated cytoplasm, indicative of glycogen storage (Fig. 2f). Nuclei were located basally, with other organelles and vesicles adjacent to apical, basal or lateral membranes, while the central part of the cells was devoid of these structures. Apical membrane of enterocytes was covered with microvilli (Fig. 2g and 2g insert). An unexpected finding was the presence of several different-sized vesicles in the intestinal lumen (Fig. 2g). The vesicles circumscribed with double membrane, resembling the cellular membrane, were filled with finely granulated, electron-lucent content. Basally, the enterocyte membrane had numerous intricate invaginations (Fig. 2h and 2h insert). Scant mitochondria were present in the basal part of the cells, where electron-dense vesicles were more abundant, corresponding to yolk vesicles. Apically, the enterocytes were connected by tight junctions, occasionally presenting more complex intercellular junctions (Fig. 2i). Abundant electron-dense tubular structures were seen subapically, below the terminal web, or in the intercellular space formed by complex junctions (Fig. 2i). Occasionally, bundles of actin fibres extending from microvilli and forming terminal web were seen (Fig. 2i). Furthermore, various MVBs or granules with multi-layered membranes, resembling myelin sheaths, were present apically, admixed with other organelles, i.e. mitochondria and Golgi apparatus (Fig. 2j and 2k).

**Fig. 3:**
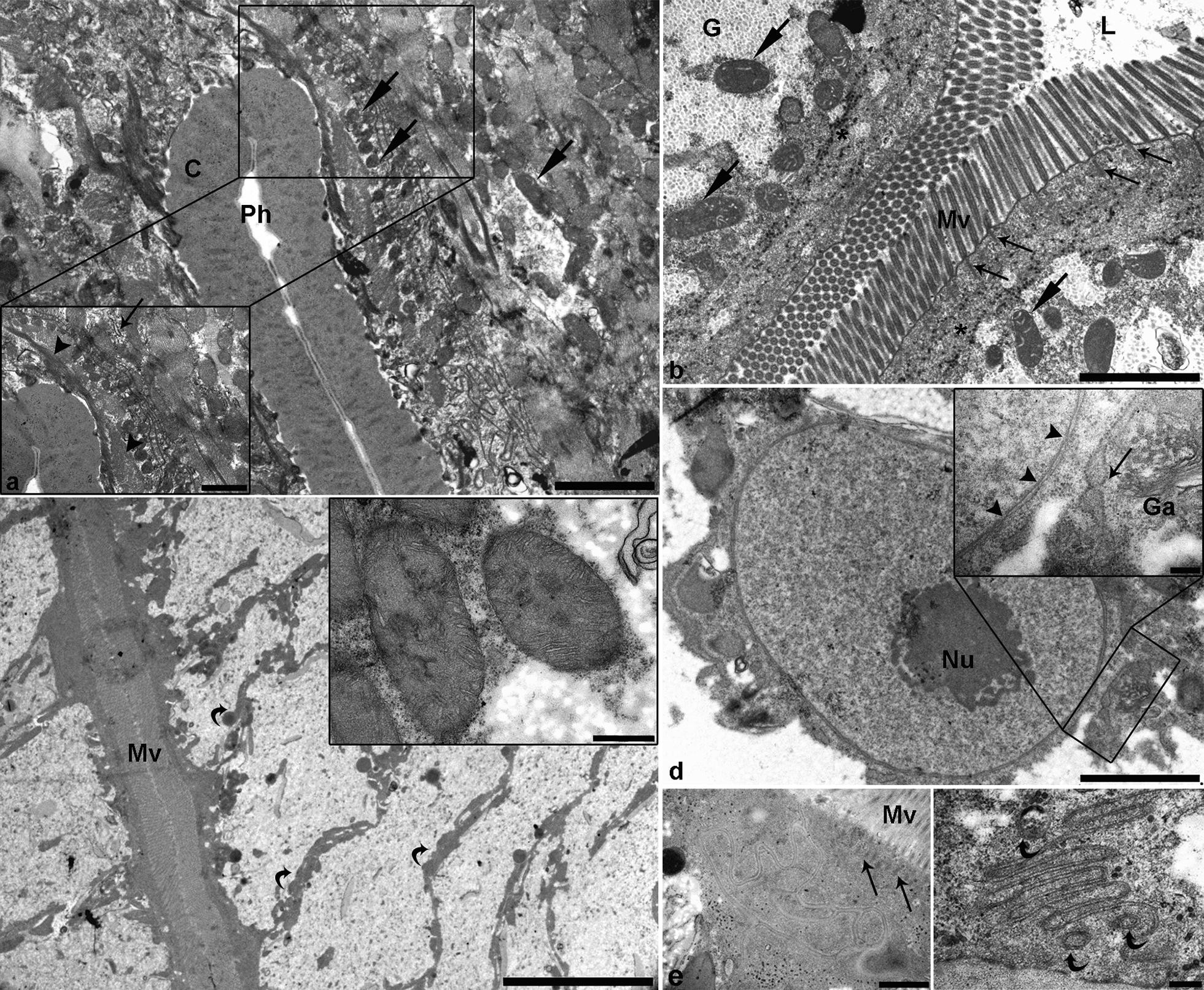
Representative electron micrographs of fourth-stage *Anisakis pegreffii* larva gut. **a** Detailed view of a pharynx with uniformly dense cuticle (C) lining the pharyngeal lumen. Numerous mitochondria (arrows) can be seen interspersed between muscle fibres. Insert: higher magnification showing densely packed muscle fibres (arrowheads), mitochondria, and rough endoplasmic reticulum (RER) (thin arrow). **b** Cross section through a collapsed intestine, showing the intestinal lumen (L) filled with finely granulated electron-lucent content. Microvilli (Mv) are directed in multiple directions, with prominent rootlets forming the terminal web (thin arrows) extending from their basal end. Numerous mitochondria (arrows) are seen subapically, surrounded by patches of granulated cytoplasm (glycogen). Note the same electron-dense tubular structures as those in L3 (asterisks). **c** Overview of spindle-shaped enterocytes with laterally located organelles (curved arrows), while the central part of the cytoplasm is empty. Insert: close-up of two large multi-cristate mitochondria. **d** Large nucleus of intestinal cell with a prominent nucleolus (Nu). Insert: detailed view of nuclear envelope with numerous nuclear pores (arrow heads). In the perinuclear space, well developed RER (long arrow) and Golgi apparatus (Ga) can be seen. **e** Complex junction between two intestinal cells. Note the long rootlets (long arrows) extending from basal ends of microvilli (Mv). **f** Deep invaginations of the basal enterocyte membrane with three small vesicles surrounded by a double membrane (curved arrows) adjacent to the invaginations. *Scale-bars*: **a**, **b**, **d**, 2 µm; **c**, 10 µm; insert in **c**, 500 nm; insert in **d**, 200 nm; **e**, 1 µm, **f**, 200 nm

As in L3, L4 pharynx was lined with uniformly dense cuticle. However, the ultrastructure of pharynx surrounding the cells differed notably in several aspects compared with the corresponding L3 cells. First, more pronounced and abundant muscle fibers radiating from cuticle lining the pharynx, and located more distally in the cell, were seen (Fig. 3a and 3a insert). Numerous mitochondria were dispersed throughout the cells between the muscle fibres. While in L3, free ribosomes were randomly scattered in the cytoplasm, in L4, the ribosomes were mostly seen forming the RER (Fig. 3a insert).

Compared with L3, L4 intestine had a tetraradiate structure when collapsed, with four lateral projections extending from the central part, lined by an enterocyte monolayer. Intestinal lumen was filled with granulated electron-lucent content; however, no vesicles as those seen in L3 intestine were present. The enterocytes presented a brush border, lined with microvilli that appeared longer and multidirectional compared with L3. Fibres extending from microvilli and forming the terminal web were more pronounced (Fig. 3b). Enterocytes cytoplasm had a coarsely granulated structure, indicating glycogen storage, but to a lesser extent compared to L3. Various vesicles and organelles were located adjacent to apical, lateral and basal membrane, leaving the central part void of organelles (Fig. 3c). A higher number of mitochondria, encompassing notably higher number of cristae, was present compared with L3 enterocytes (Fig. 3c, insert). Moreover, electron-dense vesicles, presumably containing yolk, were sparse in comparison with L3. Enterocytes contained electron-lucent vesicles, indicative of lipid storage. Large, basally located nuclei with prominent nucleoli had the RER and Golgi apparatus adjacent to them, while mitochondria were less numerous in the perinuclear space compared with L3 (Fig. 3d and 3d insert). Same apical complex junctions between enterocytes were present as in L3 (Fig. 3e), resembling interdigitations in the lateral membrane. Basal membrane of the enterocytes was invaginated as in L3. Occasionally, small vesicles circumscribed by double membrane were seen close to the invaginations (Fig. 3f).

## Discussion

### *Anisakis pegreffii* microbiota

The terrestrial mammalian immune system has co-evolved with a large number of intestinal bacteria and soil-transmitted helminths, and as helminths inhabit the bacteria-colonized intestine, they affect the microbiome as well [28–30]. Although the functional mechanisms of these effects are not clarified yet, they vary markedly with the parasite species and the intensity of infection. Likewise, bacteria-helminth associations can exist as co-evolutionary systems, wherein both cooperate to promote the establishment of each other within the intestine of their mammalian host, negatively affecting the latter. On the contrary, bacteria can have a beneficial effect on the host and negative implications for the nematode, such as in the case of protective probiotic bacteria [10, 31].

This first assessment of microbiota in larval and adult *A. pegreffii* inhabiting striped dolphins (*S. coeruleoalba*) in the Adriatic Sea revealed an ontogenic shift in the parasite’s microbial communities, in terms of both alpha diversity (number and proportion of species) and dissimilarity between communities (beta diversity) [32]. The relatively low dissimilarity indices in the three evaluated groups (gut, uterus, larvae) indicated similar microbiota communities in terms of nestedness and species turnover [33], the exceptions being the microbiota evenness between the adult and larvae microbiota (Pielou’s distance), and the dissimilarity between the adult gut and larvae microbiota (Jaccardʼs distance). The species richness was the highest in the adult gut, followed by the larvae and then the adult uterus, whereas the evenness was the highest in the adult uterus, followed by the adult gut and then the larvae. This could be explained by the ongoing microbiota dynamics usually observed in the host stomach chambers, which colonize and consequently affect the number and proportion of microbiota present in the gut of adult and larval *A. pegreffii*. In contrast, the uterus in *A. pegreffii* represents the most steady-state microbiota environment among the three groups, apparently uninfluenced by the host microbiota. Larval stages seem to share microbiota similar to that in the uterus and gut (when difference was tested among the three groups), suggesting that a part of the larval microbiota originates from the vertical transfer from the gravid uterus to the eggs, and persists throughout all consecutive larval moultings. Such transfer has been reported for *Weissella*/*Leuconostoc* complex that inhabit all life-cycle stages including eggs, of the ruminant nematode *Haemonchus contortus* [34].

The other part of the L3 microbiota in *A. pegreffii* originates from the larval settlement in the host gut, and its consequent colonisation by the dolphin’s indigenous microbiota, although L3 apparently do not actively feed at this point. Therefore, an L3 species richness similar to that of the adult gut, with an uneven proportion of species, is indicative of a system still in perturbation, depicting the succession of prelarval uterus-derived microbiota by cetacean gastro-intestinal microbiota.

After being expelled in the environment from the *A. pegreffii* uterus, larvae moult ensheathed within the egg reach the first intermediate host. Alternatively, the larvae reach the crustacean exsheated from the egg (see [4]). *In vitro*, it takes approximately 3.3 months for *Anisakis* spp. to develop from the egg to L4, of which 3–4 days are necessary for the moulting of L3 to L4 [7]. During this timeframe, the larvae experience different environments, e.g. the sea column, euphausiid pseudocoelom and fish visceral cavity; however, in the final host, analyzed infective larvae show communities similar to those of adult *A. pegreffii*, both in uterus, suggesting vertical transmission, and in the gut, the latter affected by the host microbiota. Unfortunately, we were not able to sample the host microbiota, but relied on a previously published study for comparison [35]. Interestingly, the definitive host-specific *Cetobacterium* (*Fusobacteriaceae*) does not have the highest occurrence in the adult *A. pegreffii* gut, as would have been expected, but rather in the non-feeding L3, suggesting that larvae are instantly colonized by *Cetobacterium* upon arrival in the cetacean gut. Afterwards, upon completion of the last moult, *A. pegreffii* microbiota shifts from *Cetobacterium* dominance towards *Mycoplasma*, with the latter dominating the adult *A. pegreffii* gut, as well as the striped dolphin stomach chambers [35]. Godoy-Vitorino et al. [35] observed Tenericutes order Mycoplasmatales (~43%), followed by Firmicutes, in the dolphin stomach. At the genus level, these were represented by *Peptostreptococcaceae incertae sedis* (~ 40%), *Ureaplasma* (~ 36%), *Mycoplasma* (~ 7%) and *Helicobacter* (4%), and at species level by *Helicobacter heilmannii*, *Mycoplasma*, *Ureaplasma*, and *Paeniclostridium sordellii*, all qualitatively congruent to the taxa observed in the adult *A. pegreffii*. The most striking difference compared with the dolphin stomach microbiota was in the abundance of specific taxa. Adult *A. pegreffii* microbiota was dominated by *Mycoplasma*, whereas the dolphin stomach showed more species-rich community: *Firmicutes incertae sedis*, *Ureaplasma*, *Mycoplasma*, *Helicobacter*, *Photobacterium*, *Flavobacterium*, *Cetobacterium* and *Actinobacillus*, in decreasing order of abundance. So far, Mycoplasma have been typically found in omnivorous fish, as well as in wild and aquaculture-reared Atlantic salmon in Scotland, enriching the core microbiota of salmon throughout its life-cycle [36].

In addition, 40% of *A. pegreffii* larvae were dominated by Gram-negative *Cetobacterium*, rather than *Mycoplasma*. Such discrepancy could have arisen either by the environmental properties of the two geographically remote dolphin stranding sites that we compared (e.g. the Atlantic Ocean, close to Algarve, Portugal in [35], and the Adriatic Sea in this study), or by intrinsic balancing in the adult *A. pegreffii* that manipulates the shift from the cetacean-derived communities, as found in L3, towards an adult-specific community structure different from that of its host. However, the study results could have been affected by several variables, which are difficult to control in the case of sampling of stranded cetaceans: the collection of analyzed adult *A. pegreffii* originated only from a single host, which in turn, was geographically distant from the host infected by analyzed larval stages; the unfeasibility of sampling host microbiota, as unlike the nematodes, the animals were not alive; and − 80 °C storing and soaking of L3 in levamisole-gentamicin prior to DNA extraction, rather than their bleaching.

### Rare bacteria and *Brucella* spp. in *A. pegreffii*

The possibility that the parasitic nematode is a vehicle transmitting a pathogen found in its microbiota to the parasitized host has been already noted in marine cetacean-parasite system. The cetacean filarian nematode *Parafilaroides vitulina richardsi* [37–40] is colonized by a zoonotic bacterium *Brucella ceti*, pathogenic for both the cetacean host and humans. The bacterium has been isolated from lungworm uterine tissues, implying that the nematode should be considered a potential means of *Brucella* transfer in marine mammals, as well as to humans exposed to infected marine mammals [41].

The rare bacterial species found in *A. pegreffii* account for up to 8.99% of its microbial flora (larvae, gut, uterus), including indigenous, opportunistic and pathogenic bacteria. These included genera *Clostridium* (*sensu stricto*) 1, *Enterococcus*, *Escherichia-Shigella*, *Helicobacter*, *Peptostreptococcus*, *Photobacterium*, *Pseudomonas*, *Staphylococcus*, *Ureaplasma* and *Vibrio*. Although pathogenic, the latter two have been previously isolated from asymptomatic bottlenose dolphins [35, 42], and their effect on the host remains unknown.

In contrast, marine *Brucella* spp. have been recognized as one of the most important pathogens shaping the cetacean populations, as they cause severe clinical and pathological symptoms, including abortions, male infertility, neurobrucellosis, cardiopathology, bone and skin lesions, and ultimately, stranding and death [43]. *Brucella ceti* has been cultured or detected by PCR in many cetacean species, including dolphins and whales [44, 45]. Three different groups of strains are known based on their preferred host, certain bacteriological properties and specific genetic traits: strains isolated from short-beaked common dolphin (ST23), harbour porpoise (ST26) and humans (ST27). It is believed that the strains from dolphins and porpoise are not infectious towards humans due to variable bacteriological testing results on terrestrial animals. However, evidence suggests that this classification is incorrect, as Cvetnić et al. [43] have isolated *Brucella ceti* ST27 strain from a dolphin stranded on the Croatian coast. Isolate ST26 has been detected in an adult female common minke whale (*Balaenoptera acutorostrata*) stranded in 2014 in Scotland, from a large abscess that extended from the pharyngeal region to the cranial inlet of the thoracic cavity, measuring one meter in length [46]. In addition, the same strain induced a moderate-to-severe meningoencephalitis in a striped dolphin stranded in 2012 on the Tyrrhenian coast, Italy [47].

A study on microbiota in both, a striped dolphin stranded in Portugal [35], and the one in Adriatic Sea, as presented herein, failed to detect any member of *Brucellaceae*.

### Ultrastructure of L3 and L4 gastrointestinal tract

The alimentary tract of L3 and L4 stages of *A. pegreffii* is a simple tube that conforms to the general structure of nematode gut, composed of mouth, pharynx, intestine and rectum, the only exception being the short cylindrical ventriculus, which is also found in other members of Ascaridoidea [48].

When observed under a light microscope, the only difference regarding the intestine between the two larval stages was in its triradiate appearance when collapsed in L3, compared with a tetraradiate in L4. The tetraradiate structure of collapsed L4 intestine indicates that it could assume a larger diameter, i.e. a larger volume when filled, and therefore accommodate larger amounts of food when larvae start feeding actively. More evident differences, which indicate a transition from apparently non-feeding L3 to actively feeding L4, were seen at the ultrastructural level, most notably in the pharynx and intestine. The first was in the number of muscle fibres and mitochondria in the nematode pharynx surrounding the cells, which is highly variable in structure and function across taxa, and acts as a muscular pumping organ and that is also the only apparently motile part of the nematode alimentary tract [49]. An increase in number and density of muscle fibres would, therefore, increase the motility and pumping power of the pharynx, consistent with the transition to active feeding. Furthermore, increased contraction and pumping activity of pharynx represent a higher energy demand, further supported by an increase in the number of mitochondria in L4 pharynx compared with L3. Mitochondria represent the powerhouse of cells, where energy production occurs *via* an electrochemical gradient that serves as a driving force for ATP synthesis [50]. An additional feature that caters to the higher energy demand and facilitates pharyngeal movement in L4 is the transition from oligo-cristate mitochondria, as seen in L3, to multi-cristate ones. Cristae are infoldings of the mitochondrial inner membrane on which the electrochemical gradient is established, and serve to increase the membrane surface and thus enhance the capacity for oxidative phosphorylation [50]. Moreover, the size and shape of the cristae likely regulate the energy output of oxidative phosphorylation [51]. Therefore, increase in the size and number of mitochondrial cristae and mitochondria promote the transition to L4 active feeding. Lastly, the organisation of ribosomes into rough endoplasmic reticulum (RER) in L4 pharyngeal cells compared with mostly free ribosomes in L3 pharyngeal cells, further supports a higher metabolic activity of the former.

As mentioned earlier, the only noticeable difference in the intestine between the L3 and L4 of *A. pegreffii* under a light microscope was the number of projections of collapsed intestine, although both organs are encompassed by a single enterocyte monolayer. This would suggest that adults show growth and differentiation of enterocytes, rather than cellular division and formation of new cells, as is the case in other nematodes [52]. This was confirmed at the ultrastructural level in the cellular organization of enterocytes. Although enterocytes of both larval stages had a coarsely granulated cytoplasm, indicative of glycogen storage, it was less abundant in L4 compared with L3. At the same time, electron-lucent vesicles, representing lipid droplets, were present solely in L4 enterocytes. While glycogen is essential for the anaerobic pathway of energy generation, lipids are degraded by oxidative metabolism [53]. Third-stage larvae of *Anisakis* spp. live encysted on serous membrane of visceral organs or musculature of their paratenic hosts [1], where oxygen supply can be limited. On the other hand, higher larval stages, and in turn, adults, live in stomachs of their final hosts [1], where oxygen supply is sufficient, and parasites can rely on aerobic metabolism. Similar findings were also observed in other nematode species, whose larvae live in a low-oxygen environment such as haemocoel of insects [1]. Therefore, change in the energy storage molecules further supports the transition from non-feeding L3 to feeding L4. Furthermore, increase in the number of mitochondria, organisation of ribosomes into RER, and its localisation to perinuclear space along with Golgi apparatus, together confirm the higher metabolic activity of enterocytes.

In both L3 and L4, enterocytes were encompassed by microvilli, which appeared longer and multidirectional in L4, which likely facilitate the food movement through intestine. Rootlets extending from microvilli into the apical cytoplasm and forming the terminal web were more pronounced in L4. In vertebrates, these rootlets are formed predominantly by actin filaments, interconnected by fine fibrils partly composed of myosin [54]. It is likely that same structural organization exists in invertebrates, in which case, increase in rootlet size would provide structural support to larger microvilli in L4, while interconnecting myosin fibrils might positively affect their motility [54]. Complex junctions were seen subapically in both L3 and L4 enterocytes; however, in L4 enterocytes these junctions were also present along the lateral membranes and resembled interdigitations connecting the epithelial cells. Presence of such junctions in lateral membranes could provide more structural support for expanding the intestine when large amounts of food enter the intestine.

In both larval stages, prominent invaginations of basal enterocyte membrane were seen. Such infoldings have been previously observed [52] and suggest a way of compensation in cases of inadequate transport processes between lateral cell membranes [5]. Indeed, several double-membraned vesicles were observed near such infoldings in the L4 intestine. However, these infoldings and vesicles might also serve as transcoelomatic transport between enterocytes and other nematode organs, as was suggested for similar structures in adult *Litomosoides chagasfilhoi* [55].

One striking finding in the L3 intestine was the presence of several vesicles in the intestinal lumen, whereas none were found in the L4 intestine. Although L3 represent apparently non-feeding stages, any content found within the L3 intestine might originate from passive intake during migration through the tissues of their paratenic hosts. Lack of similar findings in L4 intestine is likely the result of omission during sectioning, or alternatively, digestion by the time of sampling, which might also explain the presence of finely granulated electron-lucent in L4 intestine. Furthermore, several vesicles surrounded by multi-layered membranes, resembling myelin sheaths, were present in L3 enterocytes. Similar vesicles were also reported in the pharyngeal glands of L3 *Nippostrongylus brasiliensis*, and were suggested to protect the nematode tissue from possible histolytic activity of their content [56]. Function of these vesicles in L3 *A. pegreffii* remains unknown; however, it is possible that they also contain histolytic/digestive enzymes, with the stimulus for their synthesis arising from the vesicles found in the intestine.

## Conclusions

*Anisakis pegreffii* showed an ontogenic shift in the microbial communities, from actively non-feeding infective L3 towards the parasitizing adult forms, both in terms of alpha and beta diversity. Qualitative and quantitative composition of the L3 microbiota suggests an ongoing perturbation, owing to the larval propagation within gastric chambers of the final host. Although no striking differences were observed between the L3 and L4 *A. pegreffii* larval gut, several notable ultrastructural differences were detected, indicating the transition between these two stages. However, to obtain a deeper insight into these differences, especially their functional significance, other techniques, e.g. cytochemistry, should be employed.

## Additional files

**Additional file 1: Table S1.** Summary of *16S* rRNA microbiota samples from the *A. pegreffii*, sampled from larval (L3; *n* = 10) and adult stages (uterus; *n* = 5, gut; *n* = 5). **Table S2.** Summary of the final read counts of microbiota isolated from *A. pegreffii* L3 (larva) and adult uterus (uterus) and gut (gut).

**Additional file 2: Figure S1.** Venn diagram showing the distribution of a total of 230 distinct sequence features, identified across all samples of microbiota from *A. pegreffii* (larva, adult uterus and gut).

**Additional file 3: Figure S2.** The effect of selected sequence depth on measured indices was evaluated for each index (**a** Shannonʼs; **b** Pielou’s evenness; **c** Faith’s) by plotting the index calculated per microbiota sample of *A. pegreffii* at two rarefaction levels (optimal of 20,000 and minimal).

**Additional file 4: Figure S3.** Representative graph of rarefaction plots (observed OTUs *vs* sampling depth) for each *A. pegreffii* sample (**a**) and group (larva, gut, uterus) (**b**).

**Additional file 5: Figure S4.** The trend of each alpha diversity index [Shannonʼs (**a**, **b**), Pielou’s evenness (**c**, **d**), Faith’s (**e**, **f**)] per *A. pegreffii* developmental stage [larva, adult (**a**, **c**, **e**)] and sample [larva, gut, uterus (**b**, **d**, **f**)], at rarefaction of 20 k reads and minimal sampling depth. Asterisk (*) denotes significant difference, as tested by Kruskal-Wallis test (*H* = 1.5473, *df* = 17, *P* = 0.000734).

**Additional file 6: Figure S5.** Two-dimensional relationships between beta diversity indices [Bray-Curtis (**a-c**), Jaccardʼs distance (**d-f**), unweighted Unifrac (**g-i**), and weighted Unifrac distance (**j-l**)] for microbiota of *A. pegreffii* adult gut and larvae, analyzed using PCA (**a**, **d**, **g**, **j**), CCA (**b**, **e**, **h**, **k**) and PCoA (**c**, **f**, **i**, **j**).

**Additional file 7: Figure S6.** Differential taxonomy analysis of microbiota of *A. pegreffii* adult gut and larvae, calculated at the phylum (**a**) and class level (**b**).

**Additional file 8: Figure S7.** Differential taxonomy analysis of microbiota from *A. pegreffii* adult gut and larvae, calculated at the family level.

**Additional file 9: Figure S8.** Differential taxonomy analysis of microbiota of *A. pegreffii* adult gut and larvae, calculated at the genus level.

## Data Availability

The dataset supporting the conclusions of this article is included within the article and its additional files. Obtained sequences were deposited in the BioProject database (https://www.ncbi.nlm.nih.gov/sra) ID PRJNA512895, with accession numbers SAMN10690278–SAMN10690297.

## Abbreviations

*16S* rRNA: 16S ribosomal RNA
ALDEx: ANOVA-like differential expression analysis
CCA: canonical correspondence analysis
DADA2: divisive amplicon denoising algorithm
*Hinf*I: *Haemophilus influenzae* I
ITS: internal transcribed spacer
L3: third-stage larva
L4: fourth-stage larva
mtDNA *cox*2: mitochondrial DNA cytochrome *c* oxidase subunit 2
MVBs: multivesicular bodies
OTU: operational taxonomic units
PCA: principal components analysis
PCoA: principal coordinates analysis
QIIME2: quantitative insights into microbial ecology 2
RER: rough endoplasmic reticulum
RFL-PCR: restriction fragment length-PCR
