# Supplementary material for: Microbiota and gut ultrastructure of Anisakis pegreffii isolated from stranded cetaceans in the Adriatic Sea
Source: Parasit Vectors. 2019 Jul 30;12:381. doi: 10.1186/s13071-019-3636-z (PMC6668197; doi:10.1186/s13071-019-3636-z)
Supplement: Supplementary file 1 — Additional file 1: Table S1. Summary of 16S rRNA microbiota samples from the A. pegreffii, sampled from larval (L3; n = 10) and adult stages (uterus; n = 5, gut; n = 5). Table S2. Summary of the final read counts of microbiota isolated from A. pegreffii L3 (larva) and adult uterus (uterus) and gut (gut). [file 13071_2019_3636_MOESM1_ESM.docx]

**Additional file 1: Table S1.** Summary of 16S microbiota samples from the *Anisakis* sp. nematode, sampled from larval (L3; n=10) and adult stage, the latter subsequently divided into uterine (n=5) and gut (n=5) isolates, with their codes and sequencing parameters.

| **sample ID** | **barcode sequence** | **linker primer sequence** | **barcode name** | **reverse primer** | **stage** | **sample** |
| --- | --- | --- | --- | --- | --- | --- |
| 411b1C | GAGAGTGT | GTGCCAGCMGCCGCGGTAA | 515Fbar1 | GGACTACVSGGGTATCTAAT | adult | gut |
| 411b2U | GAGATCAG | GTGCCAGCMGCCGCGGTAA | 515Fbar2 | GGACTACVSGGGTATCTAAT | adult | uterus |
| 411c1C | GAGATCTC | GTGCCAGCMGCCGCGGTAA | 515Fbar3 | GGACTACVSGGGTATCTAAT | adult | gut |
| 411c2U | GAGATGAC | GTGCCAGCMGCCGCGGTAA | 515Fbar4 | GGACTACVSGGGTATCTAAT | adult | uterus |
| 411d1C | GAGATGTG | GTGCCAGCMGCCGCGGTAA | 515Fbar5 | GGACTACVSGGGTATCTAAT | adult | gut |
| 411d2U | GAGTACAG | GTGCCAGCMGCCGCGGTAA | 515Fbar6 | GGACTACVSGGGTATCTAAT | adult | uterus |
| 411e1C | GAGTACTC | GTGCCAGCMGCCGCGGTAA | 515Fbar7 | GGACTACVSGGGTATCTAAT | adult | gut |
| 411e2U | GAGTAGAC | GTGCCAGCMGCCGCGGTAA | 515Fbar8 | GGACTACVSGGGTATCTAAT | adult | uterus |
| 411f1C | GAGTAGTG | GTGCCAGCMGCCGCGGTAA | 515Fbar9 | GGACTACVSGGGTATCTAAT | adult | gut |
| 411f2U | GAGTCACT | GTGCCAGCMGCCGCGGTAA | 515Fbar10 | GGACTACVSGGGTATCTAAT | adult | uterus |
| 419a | GAGTCAGA | GTGCCAGCMGCCGCGGTAA | 515Fbar11 | GGACTACVSGGGTATCTAAT | larva | larva |
| 419b | GAGTCTCA | GTGCCAGCMGCCGCGGTAA | 515Fbar12 | GGACTACVSGGGTATCTAAT | larva | larva |
| 419c | GAGTCTGT | GTGCCAGCMGCCGCGGTAA | 515Fbar13 | GGACTACVSGGGTATCTAAT | larva | larva |
| 419d | GAGTGACA | GTGCCAGCMGCCGCGGTAA | 515Fbar14 | GGACTACVSGGGTATCTAAT | larva | larva |
| 419e | GAGTGAGT | GTGCCAGCMGCCGCGGTAA | 515Fbar15 | GGACTACVSGGGTATCTAAT | larva | larva |
| 419f | GAGTGTCT | GTGCCAGCMGCCGCGGTAA | 515Fbar16 | GGACTACVSGGGTATCTAAT | larva | larva |
| 419g | GAGTGTGA | GTGCCAGCMGCCGCGGTAA | 515Fbar17 | GGACTACVSGGGTATCTAAT | larva | larva |
| 419h | GAGTTCAC | GTGCCAGCMGCCGCGGTAA | 515Fbar18 | GGACTACVSGGGTATCTAAT | larva | larva |
| 419i | GAGTTCTG | GTGCCAGCMGCCGCGGTAA | 515Fbar19 | GGACTACVSGGGTATCTAAT | larva | larva |
| 419j | GAGTTGAG | GTGCCAGCMGCCGCGGTAA | 515Fbar20 | GGACTACVSGGGTATCTAAT | larva | larva |

**Additional file 1: Table S2.** Summary of the final read counts after all pre-processing steps of microbiota isolated from *Anisakis pegreffii* L3 (larva) and adult uterus (uterus) and gut (gut).

| **sample ID** | **sample** | **total input reads** | **total retained reads** | **reads truncated** | **reads too short after truncation** | **reads exceeding maximum ambiguous bases** |
| --- | --- | --- | --- | --- | --- | --- |
| 411b1C | gut | 51001 | 50854 | 145 | 36 | 111 |
| 411b2U | uterus | 45117 | 44997 | 118 | 26 | 94 |
| 411c1C | gut | 40348 | 40246 | 96 | 14 | 88 |
| 411c2U | uterus | 73554 | 73346 | 202 | 44 | 164 |
| 411d1C | gut | 59877 | 59721 | 147 | 46 | 110 |
| 411d2U | uterus | 39479 | 39376 | 104 | 20 | 83 |
| 411e1C | gut | 41949 | 41826 | 96 | 25 | 98 |
| 411e2U | uterus | 41718 | 41593 | 116 | 30 | 95 |
| 411f1C | gut | 39988 | 39872 | 104 | 24 | 92 |
| 411f2U | uterus | 40051 | 39936 | 103 | 22 | 93 |
| 419a | larva | 50833 | 50716 | 132 | 25 | 92 |
| 419b | larva | 46736 | 46582 | 128 | 30 | 124 |
| 419c | larva | 47672 | 47549 | 121 | 25 | 98 |
| 419d | larva | 47305 | 47164 | 123 | 25 | 116 |
| 419e | larva | 49521 | 49338 | 142 | 23 | 160 |
| 419f | larva | 41240 | 41122 | 123 | 31 | 87 |
| 419g | larva | 48674 | 48564 | 100 | 26 | 84 |
| 419h | larva | 48395 | 48249 | 144 | 34 | 112 |
| 419i | larva | 136495 | 136183 | 339 | 73 | 239 |
| 419j | larva | 260484 | 259839 | 506 | 271 | 374 |
